# Reviewing the review

**DOI:** 10.4102/sajid.v40i1.786

**Published:** 2025-12-20

**Authors:** Mark F. Cotton, Andrew C. Whitelaw, Ute Hallbauer, Colleen Bamford, Wolfgang Preiser, Nishi Prabdial-Sing, Nicola Page

**Affiliations:** 1 Family Center for Research with Ubuntu, Faculty of Medicine and Health Sciences, Tygerberg Hospital and Stellenbosch University, Cape Town, South Africa; 2 Division of Medical Microbiology, Faculty of Medicine and Health Sciences, Stellenbosch University, Cape Town, South Africa; 3 National Health Laboratory Service, Tygerberg Hospital, Cape Town, South Africa; 4 Department of Paediatrics and Child Health, University of the Free State, Bloemfontein, South Africa; 5 Africa Unit for Transdisciplinary Health Research, Faculty of Health Sciences, North-West University, Potchefstroom, South Africa; 6 Division of Medical Microbiology, University of Cape Town, Cape Town, South Africa; 7 Division of Medical Virology, Faculty of Medicine and Health Sciences, Stellenbosch University, Cape Town, South Africa; 8 Centre for Vaccines and Immunology, National Institute for Communicable Diseases, Johannesburg, South Africa; 9 Department of Medical Virology, University of Pretoria, Pretoria, South Africa; 10 National Institute for Communicable Diseases, National Health Laboratory Service, Johannesburg, South Africa

The *Southern African Journal of Infectious Diseases* (*SAJID*) publishes manuscripts on infectious diseases especially those relevant to our region. With a Web of Science impact factor of 1.3 for 2024, *SAJID* is an appropriate journal for both emerging and established researchers. The integrity and excellence of our journal’s output depend on the quality of the submitted manuscripts and the quality of the peer review process. For manuscript quality, we rely on the authors. While most work is usually undertaken by the first author, the supervision and input provided by the senior authors are vital, especially for manuscripts where the first author is a new or emerging researcher.

The first scientific journals, *Journal de sçavans* and *Philosophical Transactions* of the Royal Society, were published in 1665, without peer review.^1,2^ The history of pre-publication peer review began in 1731. The editor of *Medical Essays and Observations*, published by the Royal Society of Edinburgh, submitted essays to individuals he considered most suitable to review. The journal stated that peer review did not guarantee truthfulness or accuracy, which depended on the authors. The adoption of peer review was somewhat haphazard. The Royal Society established a prepublication review committee to determine whether a manuscript should be published in 1752.^3^ The *Lancet* considered peer review unimportant until 1976, and the *Journal of the American Medical Association* mainly used internal review and only occasionally outside experts until the mid-1950s.^4^ The *British Medical Journal*, however, sent all submitted manuscripts to a recognised expert, starting in 1893. Only since the late 20th century was peer review adopted by most biomedical journals.^5^ We consider it of utmost importance.

The *SAJID* uses a double-blinded peer review process where the identities of both authors and reviewers are concealed to avoid bias. We require a minimum of two reviewers per submission. Once an article passes this review process, often supplemented by additional input from the editorial team, the manuscript is published and contributes to scientific knowledge. A published manuscript not only represents the authors’ hard work but also the time and effort spent by the reviewers, editorial team and journal administrative staff.^6^

Identifying reviewers is a major challenge for *SAJID* as for most scientific journals. This difficulty is exacerbated by the plethora of new journals. The directory of open access journals currently has 398 indexed medical journals. Overall, from 2003 to 2023, there has been a nearly 200% increase in citable research documents from 1 515 000 to 4 793 000.^7^ Reviewer fatigue is now a widely recognised problem.^8^ We have had the experience of searching for and asking up to 10 people to review a publication. Potential reviewers have 2 weeks to respond to an invitation from *SAJID*. Even though difficult, we encourage reviewers to respond sooner so that we can invite someone else if they decline. Responding either late or not at all significantly increases the overall time taken to review a manuscript to completion, which is currently 92 days on average. Fortunately, most reviews are good and useful, improving the quality of the manuscripts. As reviewing a manuscript requires between 3 and 8 h of work, depending on its length and complexity, it is challenging to integrate this task into one’s daily professional life. Most reviews are conducted by colleagues in academic practice who have multiple competing obligations. These include, in addition to busy professional lives, their own research projects, seeking funding and writing manuscripts. With the proliferation of medical journals, one can be easily overburdened with requests. Some members of the *SAJID* editorial team receive several review requests per week from different journals, with each acceptance carrying its own burden of time management.

What are the benefits of reviewing for *SAJID*? We believe there are many. Firstly, one is advancing locally relevant knowledge about infectious diseases. Secondly, one is growing the capacity of emerging and established researchers to communicate their findings. Thirdly, reviewers increase their own knowledge and learn about academic activities in infectious diseases. Fourthly, reviewers are eligible for continuing education units (CEUs) as part of continuing professional development (CPD).^9^ Fifthly, peer review activities are increasingly recognised as evidence of scholarly activity and should be included in one’s curriculum vitae. We welcome their inclusion in academic performance assessments by universities and hope this will expand. Open Researcher and Contributor ID (ORCID) allows curating one’s reviewing activity. With a planned upgrade in process, *SAJID* will soon export reviewer activity to ORCID. Some journals already report reviewing activity to ORCID.

The editorial team are extremely grateful to all reviewers who accept invitations and contribute to the journal. We encourage you to accept invitations when the topic is within your field of expertise and contribute to the ongoing improvement of the research published in the journal. Should you find yourself unable to comply with a request to review or the topic does not match your area of expertise, we are grateful for suggestions of whom might be a suitable reviewer.

Mark F. Cotton (Editor-in-Chief)

Andrew Whitelaw (Deputy Editor)

Ute Hallbauer (Deputy Editor)

Colleen Bamford (Deputy Editor)

Wolfgang Preiser (Section Editor)

Nishi Prabdial-Sing (Section Editor)

Nicola Page (Section Editor)
